# EFFECTS OF UPPER LIMB VIBRATORY STIMULATION TRAINING ON MOTOR SYMPTOMS IN PARKINSON’S DISEASE: AN OBSERVATIONAL STUDY

**DOI:** 10.2340/jrm.v56.19495

**Published:** 2024-02-26

**Authors:** Valentina VARALTA, Anna RIGHETTI, Elisa EVANGELISTA, Alberto VANTINI, Alessandro MARTONI, Stefano TAMBURIN, Cristina FONTE, Ilaria A. DI VICO, Michele TINAZZI, Andreas WALDNER, Alessandro PICELLI, Mirko FILIPPETTI, Nicola SMANIA

**Affiliations:** 1 Neuromotor and Cognitive Rehabilitation Research Center, Section of Physical and Rehabilitation Medicine, Department of Neurosciences, Biomedicine and Movement Sciences, University of Verona; 2 Neurorehabilitation Unit, University Hospital of Verona; 3 Section of Neurology, Department of Neurosciences, Biomedicine, and Movement Sciences, University of Verona; 4 Neurology Unit, USD Parkinson e Disturbi del Movimento, University Hospital of Verona, Verona; 5 Department of Neurological Rehabilitation, Private Hospital “Villa Melitta”, Bolzano, Italy; 6 Canadian Advances in Neuro-Orthopedics for Spasticity Congress (CANOSC), Kingston, ON, Canada

**Keywords:** tremor, movement disorders, rehabilitation

## Abstract

**Objectives:**

Parkinson’s disease is characterized by motor and non-motor symptoms. Tremor is one of the motor symptoms that can affect manual skills and have an impact on daily activities. The aim of the current study is to investigate the effect of upper limb training provided by a specific vibratory device (Armshake^®^, Move It GmbH - Bochum, Germany) on tremor and motor functionality in patients with Parkinson’s disease. Furthermore, the training effect on global cognitive functioning is assessed.

**Design:**

An uncontrolled before-after clinical trial.

**Patients:**

Individuals with diagnosis of Parkinson’s disease, motor upper limbs deficits, and absence of dementia.

**Methods:**

Participants underwent a 3-week programme (3 times a week) and was evaluated before, after, and at 1 month follow-up by motor (Fahn Tolosa Marin Tremor Rating Scale, Unified Parkinson’s Disease Rating Scale – part III, Purdue Pegboard Test, Disability of the Arm, Shoulder and Hand Questionnaire) and cognitive (Montreal Cognitive Assessment) scales.

**Results:**

Twenty subjects are included. After treatment a statistically significant improvement in tremor, manual dexterity and activities of daily living was found. The data indicated no effects on global cognitive functioning.

**Conclusion:**

These findings suggest positive effects of vibratory stimulation training on upper limb motor symptoms in Parkinson’s disease.

LAY ABSTRACT

Tremor is one of the disabling motor symptoms in Parkinson’s disease. When tremor affects manual skills, it reduces the hand’s functionality during activities of daily living, such as writing, dressing, and eating, generating psychological consequences and reduced social participation. The objective of this study is to evaluate the effects of an upper limb vibratory rehabilitation programme on tremor and motor functionality in subjects with Parkinson’s disease. Twenty subjects were included and underwent a 3-week programme (3 times a week) using a specific device ((Armshake^®^, Move It GmbH - Bochum, Germany) and were evaluated before, after, and 1 month follow-up using motor and cognitive scales. The findings suggest positive effects of vibratory stimulation training on upper limb motor symptoms in Parkinson’s disease, including tremor. No effects were found before-after training on cognitive performance. Based on these positive data, and given the easy use and portability of Armshake^®^, it could be indicated in the physiotherapist’s clinical practice and home-based rehabilitation programme.

Parkinson’s disease (PD) is a neurodegenerative illness characterized by motor and non-motor disorders (1). Tremor is one of the motor symptoms, together with rigidity, bradykinesia, and postural instability (2). Tremor is defined as a rhythmic, involuntary, and oscillatory movement of part of the body (3), and is observed in 80% of subjects during different conditions (action/intention, at rest, posture holding) (4). Tremor can involve the upper or lower limbs, including hands and feet, tongue, or jaw (3). Specifically, resting tremor is characterized by an amplitude between 4 and 7 Hz (5). Stressful situations, mentally demanding tasks, or dual motor tasks, typically provoke resting tremor (6), whereas under calm and idle conditions the tremor diminishes (7). Tremor is often underestimated despite being reported as the most annoying symptom (8). Furthermore, this condition, together with bradykinesia and rigidity, leads to deficits in manual skills from the earliest stage of PD (9).

The tremor usually reduces hand’s functionality during activities of daily living (ADL) (e.g. writing, dressing and eating), generating psychological consequences and reduced social participation (10, 11). Furthermore, patients with newly diagnosed and untreated PD also demonstrated impairment in handwriting, pointing, and aiming tasks (11).

At the pathophysiological level, tremor, differently from bradykinesia and rigidity, does not appear to be directly linked to dopamine deficiency in the substantia nigra (12). Deficit in finger dexterity is related to an intrinsic dysfunction of primary somatosensory cortex, which is not reversible by dopaminergic medication (13).

Rehabilitation could play an important role in the management of this symptom to minimize the undesirable effects of drug therapy and maximize functional abilities, improving the perception of self-efficacy of the patient (12).

Tremor in PD involves a complex interaction between central and peripheral mechanisms. Indeed, different approaches have been developed to reduce tremor.

An improvement in resting tremor was found after changes in mechanical conditions during externally imposed movements of a joint, or by electrical muscle stimulation (EMS) (14). EMS can be also used to reduce other upper limb symptoms modulating the peripheral reflex mechanism (15, 16). Other approaches involve motor exercises, such as hand activities (17, 18) or cycling (19), to decrease amplitude and/or frequency tremor in patients with PD (20). Physical exercise can also improve other motor symptoms (20) in the different stages of PD (21), such as motor coordination and grasp strength (17) and bradykinesia (19).

However, further research is needed to define the clinical application of a training programme focusing on the reduction of upper limb motor symptoms in patients with PD. The objective of this study is to evaluate the effects of an upper limb (UL) vibratory rehabilitation programme using a specific device (Armshake^®^, Move It GmbH - Bochum, Germany) on tremor and motor functionality in patients with PD.

## METHODS

### Study design

This uncontrolled before-after clinical trial included patients with PD recruited between January 2022 and July 2022 from the Neurorehabilitation Unit of University Hospital of Verona, Italy. The study was approved by Comitato Etico per la Sperimentazione Clinica delle Province di Verona e Rovigo (Code: 3670CESC (CESC)). All participants were informed about the study procedures and provided written informed consent before taking part in the assessment. The protocol was performed following the principles of the Declaration of Helsinki, and was registered on Open Science Framework (https://osf.io/ch8mb; DOI: 10.17605/OSF.IO/CH8MB).

### Participants and setting

Subject with diagnosis of PD according to UK Parkinson’s disease Society Brain Bank Criteria (22) were recruited. Participants presented resting tremor (assessed by Movement Disorder Society (MDS)-Unified Parkinson’s Disease Rating Scale-part III – MDS-UPDRS-III) (26), disease stage between 1 and 3 at Hoen and Yahr (H&Y) classification (determined in the “on” phase) (23), and absence of dementia (Montreal Cognitive Assessment (MoCA) > 15, 50) (24) were included. Subjects were excluded if they presented other neurological disorders or orthopaedic conditions involving the upper limbs, recent change in drug therapy, psychiatric disorders, alcohol or drug abuse, uncorrected visual or auditory deficits. The participants did not perform any type of rehabilitation in the 2 months leading up to the study. The training was performed at the neurorehabilitation gymnasium in the Neurorehabilitation Unit of University Hospital of Verona, Verona, Italy.

### Intervention and procedures

Participants underwent a 3-week one-to-one treatment, 3 times a week, for a total of 9 sessions. Each session lasted approximately 45 min, led by a physiotherapist specialized in neuromotor rehabilitation. Treatment consisted in carrying out an upper-limb exercises protocol holding the Armshake^®^ device, produced by Move it GmbH (www.move-it-med.com) (Fig. S1).

**Fig. 1:**
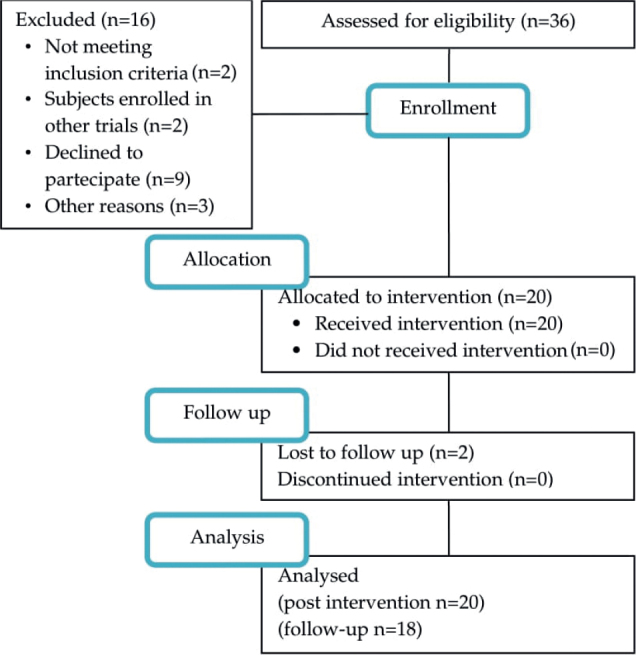
Study flow diagram.

The Armshake^®^ device has a flat part, with a rotating plate that can produce different vibration frequencies (range 2–20 Hz). The vibration frequencies are set on a tablet connected via Bluetooth to the device and is transmitted to the entire upper limb. The training session provided tasks performed with single or double upper limbs, both sitting and standing positions, holding Armshake^®^ according to the exercise protocol (Fig. 1). During the activities, participants were stimulated to focus their attention on the position and movement of their upper limbs, preserving the gripping force on the device. In addition, during tasks in the standing position, the participants were encouraged to maintain a postural stance as erect as possible, working on the coordination between upper and lower limbs and trunk.

The intervention was adapted to each individual, taking into account their level of motor and cognitive functionality. Specifically, the adaptation in terms of the difficulty of exercises was made for each session (i.e. number of repetitions, duration of the exercise, rest time between them, sit down/standing position, reducing the range of motion, preferring to maintain a position, reducing the frequency of movement).

### Variables

Motor and cognitive evaluation were performed before (T0), at the end (T1) and 30 days after the end of treatment (T2).

The global motor assessment, Unified Parkinson’s Disease Rating Scale-part III (MDS-UPDRS-part III), was conducted by a physiatrist (AM). Other motor and cognitive measures were performed respectively by a physiotherapist (AV) and a neuropsychologist (EE).

Outcome measures assessors were not involved in the rehabilitation intervention and were blinded on the timing of the assessment (i.e. T0, T1, T2). The participants were tested in the “on” state. All subjects were on dopaminergic medication.

### Primary outcome measure

The primary outcome measure was Fahn Tolosa Marin Tremor Rating Scale (FTMTRS) (25), a 21-items rating scale. It is used to quantify essential tremor severity and its impact on ADL. Each item is rated on a scale from 0 (= no tremor) to 4 points (= severe amplitude) (higher score=greater severity; range 0–144).

### Secondary outcome measures

The 3 different parts (a, b, c) in which the FTMTRS primary assessment scale is structured were considered secondary outcome measures. The FTMTRS enables assessment of: (a) tremor location and severity (items 1–9; range 0–80): FTMTRS_TR; (b) ability to perform specific motor tasks (writing, drawing and pouring, with dominant and non-dominant hands) (items 10–14; range 0–36): FTMTRS_WD; (c) patient-reported functional disability resulting from the tremor (speaking, eating, drinking, hygiene, dressing, writing, working and social activities) (items 15–21; range 0–28): FTMTRS_ADL. Based on the training target the study also calculated a specific score for UL (FTMTRS_TR_UL) summing the items 5 and 6 regarding at rest, postural holding, and action/intention tremor (right and left hand) (range 0–24) (higher score=greater severity). Based on the training target, the current study also calculated a UL-specific score (FTMTRS_TR_UL) by summing items 5 and 6 regarding rest, postural holding, and action/intention tremor (right and left hand) (range 0–24). (Higher score=greater severity).

Other secondary motor and cognitive outcome measures were: MDS-UPDRS-part III, Purdue Pegboard Test (PPT), Disability of the Arm, Shoulder and Hand (DASH) questionnaire and cognitive test MoCA.

The MDS-UPDRS-III was used to assess global movement capacity. It consists of 18 items (each rated on a scale from 0 to 4 points) about tremor, slowness (bradykinesia), stiffness (rigidity), and balance. The total score is the sum of all items (higher score=greater severity; range 0–136) (26).

To test hand dexterity PPT is used. The current study considered: (*i*) total number of pins inserted with left, right and both hands (PPT_combined); and (*ii*) number of pieces (containing holes, pegs, washers and cylinders) assembled completely within 60 s using both hands (PPT_ASS). A higher score corresponds to better performance (27).

To investigate the subjective disability of the upper limbs, during ADL, the DASH is administered (higher score = greater severity; range 0–150) (28).

The MoCA was used to assess patient’s global cognitive status. The test includes items to investigated different cognitive abilities (visual-spatial skills, executive functions, language, orientation, attention and memory). The total score varies between 0 and 30 (higher score = greater severity) (24).

### Statistical methods

Data were analysed using Statistical Package for Social Science (IBM SPSS) version 26.0 software for Macintosh. Data distribution was determined using Kolmogorv-Smirnov and Shapiro-Wilk tests. Normal variables were analysed with 1-way analysis of variance (ANOVA) for repeated measures with within-individual factor “time” (T0, T1, T2). The other variables were analysed by Wilcoxon signed-rank (within-group comparison). Alpha level for significance was set at *p* < 0.05. Post-hoc comparisons were corrected with least-significant difference (LSD) method. To avoid bias, the statistical analysis included only the data patients who underwent the specific timing assessment.

Power analysis carried out with G*Power 3.1.9.4 software, indicated that, for 1 group being measured across 3 observations, an alpha of 0.05, a power of 0.80, and large treatment effect of 0.08, 16 observations were needed to detect a significant treatment effect. Therefore, a total of 18 participants were suitable considering a possible dropout of 15%.

### Data availability

Data associated with the paper are not publicly available, but are available from the corresponding author upon reasonable request.

## RESULTS

Twenty patients (18 males; age: 70.65 ± 8.5 years; education: 8.8 ± 3.42 years; H&Y classification: 8 participants stage 3, 5 participants stage 2 and remaining stage 1; presenting idiopathic PD (mean disease duration: 5 ± 4.62 years) were recruited from among 36 outpatients referring to the Neurorehabilitation Unit of University Hospital of Verona, Italy, between January 2022 and July 2022. The participants were allocated for upper limb motor training. No adverse events were recorded during the study. All subjects completed the training programme and T1 evaluation; 18 patients also completed follow-up assessment. The study flow diagram is shown in Fig. 1.

### Outcome data and main results

*Baseline.* Among outcome measures FTMTRS, different parts in which the FTMTRS primary assessment scale is structured which FTMTRS_TR, FTMTRS_TR_UL and FTMTRS_ADL, PPT and MoCA, scores resulted normally distributed (Kolmogorov-Smirnov and Shapiro-Wilk test, *p* > 0.05) and the analysed with parametric tests.

The remaining outcome measures (MDS-UPDRS-III, DASH, FTMTRS_WD), were analysed with non-parametric tests (Wilcoxon signed-rank test).

*Primary outcome.* One-way ANOVA for repeated measures showed statistically significant differences between pre-post treatment regard FTMTRS (F = 29.648; *p* < 0.001). This result is also maintained at follow-up (*p* < 0.001).

*Secondary outcomes.* For the outcome measures analysed with parametric tests, ANOVA showed a significant effect between pre-post treatment of the time factor regarding PPT: PPT_combined (F = 3.986; *p* = 0.028) and PPT_ASS (F = 8.624; *p* = 0.001). Specifically, a significant change in post hoc analysis emerges, which is also maintained at follow-up (PPT_combined, *p* = 0.016; PPT_ASS, *p* < 0.001).

Statistically significant differences in time factor emerged for each domain of the scale FTMTRS: FTMTRS_TR (F = 13.033; *p* = 0.001), FTMTRS_TR, FTMTRS_TR_UL (F = 9.134; *p* = 0.001), FTMTRS_ALD (F = 6.928; *p* = 0.003). The results for outcome measures analysed with non-parametric tests are shown in Table I.

**Table I:** Group data and results for outcome measures analysed with parametric and non-parametric tests

Outcome	Pre-treatment	Post-treatment	Follow-up	Repeated measures ANOVA *p*-value	Post-hoc analysis		
					Post-treatment vs pre-treatment *p*-value (95% CI)		Follow-up vs pre-treatment *p*-value (95% CI)
*Parametric tests*							
FTMTRS (0–144), mean (SD)	21.90 (12.98)	13.90 (10.14)	12.06 (9.23)	<0.001*	<0.001 (5.26; 11.08)*		< 0.001 (6.28; 13.38)*
FTMTRS_TR (0–80), mean (SD)	7.60 (6.33)	3.95 (3.41)	3.50 (2.94)	0.001*	0.001 (1.71; 5.74)*		0.001 (1.84; 6.39)*
FTMTRS_TR_UL (0–36), mean (SD)	4.70 (3.15)	2.70 (1.95)	2.50 (1.62)	0.001*	0.001 (0.92; 3.08)*		0.008 (0.59; 3.41)*
FTMTRS_ADL (0–28), mean (SD)	4.90 (4.14)	3.40 (3.97)	3.06 (3.92)	0.003*	0.016 (0.32; 2.79)*		0.05 (0.56; 2.67)*
PPT_combined (score), mean (SD)	25.75 (8.12)	27.60 (7.46)	27.61 (8.44)	0.028*	0.036 (–3.64; –0.13)*		0.016 (–2.99; –0.35)*
PPT_ASS (score), mean (SD)	16.70 (6.01)	18.75 (6.03)	19.17 (6.43)	0.001*	0.011 (–3.39; –0.50)*		< 0.001 (–3.47; –1.31)*
MoCA (0–30), mean (SD)	22.35 (4.92)	23.00 (4.69)	23.06 (3.26)	0.468	/		/
Outcome	Pre-treatment	Post-treatment	Follow-up	Within-group comparison			
				Post vs pre- treatment, *p*-value		Follow-up vs pre-treatment *p*-value	
*Non-parametric tests*							
MDS-UPDRS-III (0–136), median (IQR)	20.00 (16.75; 38.25)	18.00 (13; 33)	18.50 (14.5; 30.75)	*0.005* *		0.028*	
DASH (0–150), median (IQR)	47.00 (36.00; 51.25)	39.00 (32.00; 43.75)	36.00 (32.00; 46.00)	<0.001*		*0.001* *	
FTMTRS_WD (0–36), median (IQR)	8.50 (7.75; 10.25)	5.00 (3.00; 8.25)	4.50 (2.25; 7.75)	<0.001*		*0.001* *	

SD: standard deviation; 95% CI: 95% confidence interval; IQR: interquartile range; FTMTRS: Fahn Tolosa Marin Tremor Rating Scale, FTMTRS_TR_UL: Fahn Tolosa Marin Tremor Rating Scale items 5+6 resting tremor+postural+kinetics upper limbs, FTMTRS_WD: Writing, FTMTRS_ADL: activities of daily living; PPT_combined: Purdue Pegboard Test dominant hand+non dominant hand+both hands, PPT_ASS: Assembly; MoCA: Montreal Cognitive Assessment; MDS- UPDRS-III: Unified Parkinson’s Disease Rating Scale part III; DASH: Disability of the Arm, Shoulder and Hand; MoCA: Montreal Cognitive Assessment;

* statistically significant (*p*<0.05).

Regarding outcome measures analysed by non-parametric tests, there was a significant change in the performance of DASH (T0-T1: Z = –3.67, *p* < 0.001), FTMTRS_WD (T0-T1: Z = –3.49, *p* < 0.001) and MDS-UPDRS-III (T0–T1: Z = –2.83; *p* = 0.005) after treatment. The improvement was maintained at follow-up (for details, see Table I). Regarding MoCA, no statistically significant differences emerged between timing (pre-post Z = –1.51, *p* = 0.13; pre-follow-up Z = –0.54, *p* = 0.59).

## DISCUSSION

The goal of this uncontrolled before-after clinical trial was to investigate the effects of a vibratory training for upper limbs on tremor in patients with PD.

After rehabilitation, this study observed a statistically significant decrease in global tremor. It is important to highlight that this improvement occurred specifically for the upper limbs and resulted in a better performance for writing and drawing abilities.

To our knowledge, the Armshake^®^ device has never been tested on PD. Therefore, it is not possible to directly compare the current data with previous studies.

However, these results seem to be in line with those obtained using electrically activated muscles approaches (14, 16, 29). These therapies induce forces able to cancel out the involuntary tremorogenic activation. Specifically, the EMS probably stimulates the antagonistic muscles during involuntary activation of agonist muscles and vice-versa (15).

Based on this assumption, the vibratory stimulation could promote a central integration of proprioceptive stimuli by the elicitation of the tonic stretch reflex and the activation of the sensitive areas of the central nervous system (30, 31). The hypothesis is that the stimulation would bypass basal ganglia circuitry, which is affected in PD, improving patients’ strength and endurance (15, 16). However, it is currently not known as the Armshake^®^ device exactly acts. Future studies could help to better understand the operating mechanisms through more in-depth assessments.

On the other hand, as demonstrated by literature data, physical exercise can reduce motor symptoms, including tremor. Specifically, hand movement activities for grasping and picking up an object (18, 32) showed a positive effect on movement disorders in patients with PD (18, 32). In agreement with these studies, these data indicated a significant improvement in global motor functioning and manual dexterity, which was maintained at follow-up.

The Armshake^®^ programme provides a specific upper limb training. It is possible to hypothesize that this treatment increases upper limb strength, resulting in improved performance to the test of dexterity. Some authors hypothesized that hand motor exercises activate the thalamus (ventral area) and putamen (33, 34). Furthermore, these neural regions are more stimulated by repetitive-rhythmic upper limb’s movements acting as proprioceptive inputs (35). This could explain the positive effect on the general symptoms of PD.

The current study indicated that the motor performance obtained by outcome measures was maintained at follow-up. This result seems to indicate that 1 month of treatment with the Armshake^®^ device is sufficient for an improvement that is maintained over time.

Data obtained from the DASH questionnaire suggest that Armshake^®^ training can also provide a benefit on a functional level in a real-life context. Indeed, the trained subjects feel less restricted by the presence of tremor while performing daily activities.

We consider this result relevant because the rehabilitation goal does not aim only at an improvement in the impairments structural and functional, but also to a reduction in activity limitations and participation restrictions (10).

Finally, considering the impact of cognitive functioning on patients with PD (36, 37), we investigated the influence of Armshake^®^ training on cognition. In agreement with studies that provided anaerobic interventions (38, 39), the current results indicated no effects of training on global cognitive functioning. We expected this result because the MoCA investigated global cognitive status and is not enough to properly highlight different cognitive functions. More accurate examination by function-specific tests would be necessary to better understand the possible effects of the Armshake^®^ device on cognitive performance. Indeed, as reported in the preview study, cognitive changes after training were found only using function-specific cognitive testing (40).

This study has some limitations. Since the patients were not tested in a medication “off” state, no conclusions can be drawn about the unmedicated state. A sample size with greater balance regarding sex and age could better validate the current results. Moreover, further investigations are needed to compare the effects of other upper limb rehabilitation approaches, or combined programmes (cognitive plus motor), or control no-treated group on motor and cognitive abilities. Using the FTMTRS is considered a possible strength of this study because it specifically evaluates tremor and can be used in all clinical contexts (25). On the other hand, it would be interesting to quantify tremor objectively measure by instrumental measures (e.g. accelerometer) as outcome. Finally, it would be useful to perform longer-term follow-up to determine how long the effects of treatment with this device are maintained.

In conclusion, these results suggest that treatment with the Armshake^®^ device could be effective in reducing tremor, improving manual dexterity and patient’s ability in their daily lives. These findings might serve as a starting point to better investigate the effects of vibratory stimulation training on motor and cognitive symptoms in patients with PD. More research is needed into use of the Armshake^®^ device in clinical practice or home use for appropriately trained patients.
